# Interplay of LncRNAs NEAT1 and TUG1 in Incidence of Cytokine Storm in Appraisal of COVID-19 Infection

**DOI:** 10.7150/ijbs.72318

**Published:** 2022-07-18

**Authors:** Safaa I. Tayel, Eman A. El-Masry, Gehan A. Abdelaal, Somaia Shehab-Eldeen, Abdallah Essa, Nashwa M. Muharram

**Affiliations:** 1 Medical Biochemistry and Molecular Biology Department, Faculty of Medicine, Menoufia University 32511, Shebin El-Kom, Egypt.; 2 Medical Biochemistry Unit, College of Medicine, Al Baha University, Al Baha 65779, Saudi Arabia.; 3 Microbiology and Immunology Unit, Department of Pathology, College of Medicine, Jouf University, Sakaka 72388, Saudi Arabia.; 4 Medical Microbiology and Immunology Department, Faculty of Medicine, Menoufia University, Shebin El Kom 32511, Egypt.; 5 Chest Department, Faculty of Medicine, Menoufia University, Shebin El Kom 32511, Egypt.; 6 Tropical Medicine Department, Faculty of Medicine, Menoufia University, Shebin El Kom 32511, Egypt.; 7 Internal Medicine Department, College of Medicine, King Faisal University, Al-Ahsaa 31982, Saudi Arabia.

**Keywords:** COVID-19, IL-6, NEAT1, TNF-α, TUG1

## Abstract

**Background**: In 2019, the coronavirus pandemic emerged, resulting in the highest mortality and morbidity rate globally. It has a prevailing transmission rate and continues to be a global burden. There is a paucity of data regarding the role of long non-coding RNAs (lncRNAs) in COVID-19. Therefore, the current study aimed to investigate lncRNAs, particularly NEAT1 and TUG1, and their association with IL-6, CCL2, and TNF-α in COVID-19 patients with moderate and severe disease.

**Methods**: The study was conducted on 80 COVID-19 patients (35 with severe and 45 with moderate infection) and 40 control subjects. Complete blood count (CBC), D-dimer assay, serum ferritin, and CRP were assayed. qRT-PCR was used to measure RNAs and lncRNAs.

**Results**: NEAT1 and TUG1 expression levels were higher in COVID-19 patients compared with controls (P<0.001). Furthermore, CCL2, IL-6, and TNF-α expressions were higher in COVID-19 patients compared to controls (P<0.001). CCL2 and IL-6 expression levels were significantly higher in patients with severe compared to those with moderate COVID-19 infection (P<0.001). IL-6 had the highest accuracy in distinguishing COVID-19 patients (AUC=1, P<0.001 at a cutoff of 0.359), followed by TUG1 (AUC=0.999, P<0.001 at a cutoff of 2.28). NEAT1 and TUG1 had significant correlations with the measured cytokines, and based on the multivariate regression analysis, NEAT1 is the independent predictor for survival in COVID-19 patients (P=0.02).

**Conclusion**: In COVID-19 patients, significant overexpression of NEAT1 and TUG1 was observed, consistent with cytokine storm. TUG1 could be an efficient diagnostic biomarker, whereas NEAT1 was an independent predictor for overall survival.

## Introduction

Coronavirus disease 2019 (COVID-19) is a worldwide pandemic with high incidence and rapid transmission rates. The current global situation reveals 508,827,830 confirmed cases and 6,227,291 deaths, with the majority of cases prevalent in Europe and the Americas and least in Africa, and a total of 11,426,994,800 vaccine doses administered 1. Coronavirus is an enveloped, positive-sense RNA virus that causes respiratory, enteric, hepatic, and neurological diseases in humans. Currently, seven human coronaviruses (HCoVs) can infect humans, and estimates suggest that 2% of the population are healthy carriers of HCoVs, with these viruses accounting for 5% to 10% of acute respiratory infections 2. COVID-19 is caused by the Severe Acute Respiratory Syndrome Coronavirus 2 (SARS-CoV-2), a beta coronavirus in group 2B with over 70% genetic similarity to SARS-CoV-1 3.

The most common clinical symptoms of severe COVID -19 are pneumonia accompanied by fever, cough, and dyspnea. According to the Chinese Center for Disease Control and Prevention, of 44,500 cases of detected infections, 80% of those patients had mild disease (no pneumonia or mild pneumonia), 14% had severe disease (dyspnea, hypoxia, or more than 50% lung involvement on imaging tests), and 5% had the acute disease (with respiratory failure, systemic shock, or multiple organ failure) 3, 4.

Severe COVID-19 syndrome is distinguished by overactive and destructive inflammation 5. COVID-19 triggers signaling cascades in response to viral contact, leading to the release of type I interferons, cytokines, and chemokines (IL-1β, IL-2, IL-6, IL-17, TNF-α, G-CSF, IFN-γ, CXCL10, CCL2 and CCL3, i.e., the cytokine storm). The severity of the disease is related to the secretion of these cytokines 6.

Long non-coding RNAs (LncRNAs) are non-coding RNAs (ncRNAs) that are endogenously expressed and are more than 200 nucleotides in length. Through various mechanisms, lncRNAs induced by viruses regulate innate immune responses to clear viral infections 7. A broad spectrum of research studies has shown that lncRNAs are involved in many biological processes, including inflammation, cellular function, and immune dysfunction, and that they play an essential role in the progression of various diseases, including viral infections 8, 9.

LncRNA nuclear paraspeckle assembly transcript 1 (NEAT1) is closely related to the immune responses, and likely contributes to the inflammatory evolution of SARS-CoV-2 infected cells 10, 11. NEAT1 predicts increased susceptibility to COPD and an increased risk of acute exacerbations and correlates positively with disease severity as well as inflammation 12. Furthermore, in sepsis-induced acute lung injury, NEAT1 enhanced cell apoptosis and inflammatory response in WI-38 cells via the miR-944/TRIM37 axis 13.

Taurine upregulated gene 1 (TUG1), also known as TI-227H, LINC00080, and NCRNA00080 is located on the human chromosome 22 autosomal long arm one region two sub-band (22 q12.2), with a total length of about 7.1 kb that was induced by taurine in developing mouse retinal cells 14. Recently, researchers have observed a significant decrease in TUG1 levels in non-small cell lung cancer (NSCLC) 15. TUG1 was found to be increased in gastric cancer, osteosarcoma, and bladder cancer, according to some studies 16-18, which is likely because lncRNAs exhibit tissue-specific expression 19 predominantly. In contrast, the function and potential mechanism of TUG1 in COVID patients have not yet been defined.

According to recent studies, uncontrolled inflammation promotes disease severity in COVID-19 in addition to direct viral damage 20. Consistent with this hypothesis, patients with severe diseases have elevated inflammatory markers, such as C-reactive protein (CRP), ferritin, D-dimer, and elevated neutrophil-to-lymphocyte ratio and inflammatory cytokines and chemokines 4. Patients with pathogenic inflammation, also known as cytokine storms, have similarities to those infected by other severe coronaviruses, such as SARS-CoV and Middle East respiratory syndrome coronavirus 21.

Interleukin-6 (IL-6) is secreted by nearly all stromal cells as well as by the immune system cells, such as B lymphocytes, T lymphocytes, macrophages, monocytes, dendritic cells, mast cells, and other non-lymphocytes, like fibroblast and endothelial cells 22. In response to a SARS-CoV-1 respiratory tract attack, IL-6 can provoke hyper-innate inflammation 23. C-C motif chemokine ligand 2 (CCL2) is involved in several processes, including immunological and inflammatory reactions, and may affect cell homeostasis and the energy needs of metabolic organs 24. Tumor necrosis factor-alpha (TNF-α) is a critical component of almost all acute inflammatory processes. TNF-α blocking has been shown to contribute to treating other autoimmune inflammatory diseases, suggesting that it could have a role in managing COVID-19 patients to reduce their organ damage 25. This study meant to discover the role of two critical lncRNAs, namely NEAT1 and TUG1 expressions in COVID-19 infections, and their relationship with cytokine storm through measuring IL-6, CCL2, and TNF-α in provocation and prognosis in COVID-19 infections.

## Subjects and methods

### Subjects

This prospective study was conducted on 80 newly diagnosed COVID-19 patients enrolled in the Chest Department at Menoufia University Hospital from June to July 2020 in collaboration with the Medical Biochemistry and Molecular Biology, Medical Microbiology and Immunology, and Tropical Medicine departments. They were randomly categorized into two groups: the severe COVID-19 group included 35 patients with severe COVID-19 infection, whereas the moderate COVID-19 group included 45 patients with moderate COVID-19 infection. The control group included 40 subjects of matched age and sex who had no history of acute infection and were tested for COVID-19 and determined to be negative.

COVID-19 was diagnosed based on clinical findings, radiologic investigation, and/or a positive result from the nasopharyngeal swab and a real-time reverse transcriptase-polymerase chain reaction (RT-PCR) for the respiratory pathogen. COVID-19 severity was categorized as mild, moderate, and severe. Patients with mild COVID-19 had symptoms but no pneumonia or hypoxia, while those with moderate COVID-19 had pneumonia but no hypoxia. Severe cases were associated with (1) dyspnea (≥ 30 breath/min), (2) low blood oxygen saturation of ≤ 93%, (3) PaO_2_/FiO_2_ ratio < 300, or pulmonary infiltration > 50% within 24-48 hours. The condition was classified as severe if any of the above criteria were met 26. Inclusion criteria: Patients diagnosed with COVID-19 based on the criteria mentioned above with suspected cytokine storm 27 based on the clinical deterioration such as (fever, tachypnea, fatigue, headache, anorexia, diarrhea, rash, arthralgia, myalgia, and neuropsychiatric manifestations) and the laboratory findings elevation above the threefold upper normal level of at least two of the following markers CRP, elevated IL6, ferritin and D-dimer levels or deterioration of oxygen levels (PaO_2_, SaO_2_) 28. Exclusion criteria: Patients with chest infections and sepsis if there is discoloration of sputum, leukocytosis, culturing of respiratory secretions such as sputum or tracheal aspirate, or blood culture revealing bacterial infections. Total survival time was calculated based on the time between enrollment in our study (blood withdrawal) and death or last recorded contact with the patient.

All patients provided written informed consent. The ethics committee of Menoufia University Hospital approved the protocol for this study, and the study was carried out in accordance with the guidelines of the Declaration of Helsinki.

### Methods

**Blood Sampling:** Ten- millilitres venous blood samples were collected from study participants; 2ml were placed in ethylenediamine tetra-acetic acid (EDTA)- tubes for a complete blood count (CBC) by Sysmex XN-10 haematology analyser (Sysmex Corporation, Kobe, Japan); 2ml of fresh blood on EDTA tubes were centrifugated at 4000 rpm for 10 min for plasma isolation for D-dimer assay (µg/ml FEU) using STA compact coagulometer (Diagnostica Stago, Asnieres, France). Serum was separated from 3ml of blood in a simple tube, coagulated for 30 min, and centrifugated at 4000 rpm for 10 min to evaluate serum ferritin (ng/ml) using the Architect i1000SR immunoassay (Abbott, Illinois, USA) and serum CRP using Mispa i2 (Agappe Diagnostics, Cham, Switzerland). The remaining 3ml of blood were placed in EDTA- tubes, centrifugated at 4000 rpm for 10 min for plasma detachment needed for RNA separation, including lncRNA, and **counted** by quantitative real-time PCR (qRT-PCR). Oxygen saturation was measured by pulse oximeter (Shenzhen IMDK Medical Technology Co., Ltd, Guangdong, China).

### Measurement of RNA and LncRNAs expressions by (qRT-PCR)

**Figure 1:**
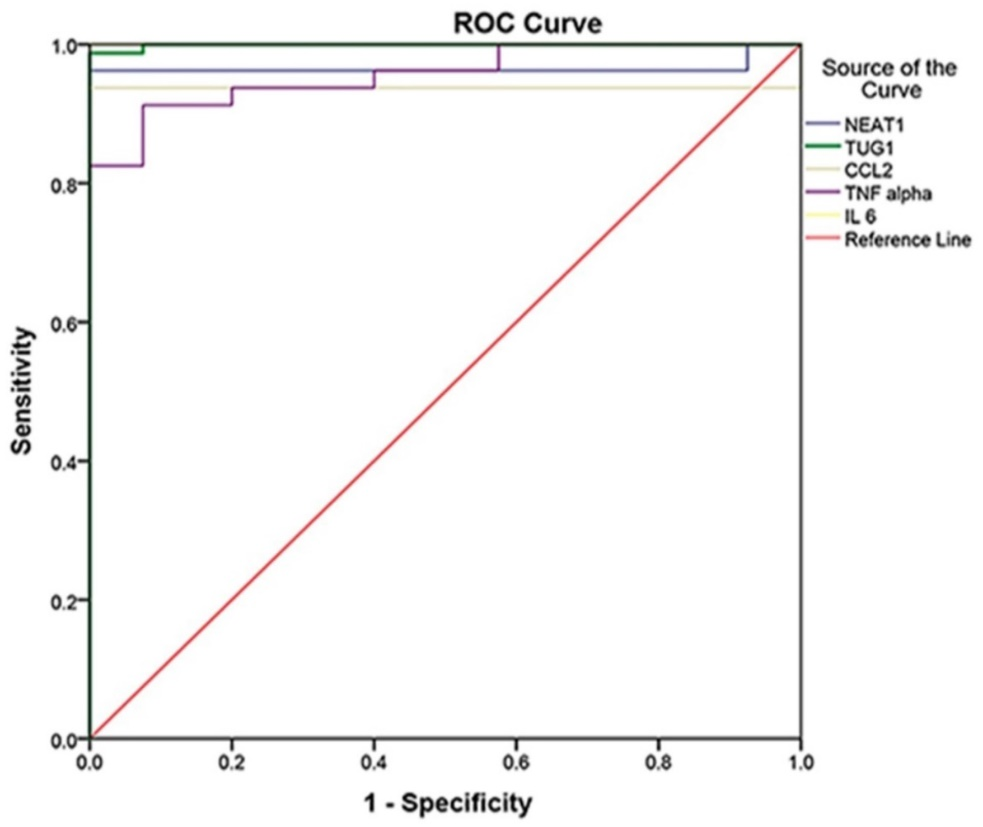
ROC Curve analysis of markers to differentiate between cases and controls (cases N = 80, controls N = 40, p<0.001), in which IL-6 was the best with 100% sensitivity, 100% specificity, and AUC = 1, followed by TUG 1 with AUC = 0.999, NEAT1 with AUC = 0.965, CCL2 with AUC = 0.938, and TNF- with AUC = 0.957.

RNA, including lncRNAs, were detached from plasma utilizing kits supplied by (miRNeasy Mini Kit, Qiagen, Germany), followed by RNA quality and purity testing with the aid of nanodrop (ND-1000 spectrophotometer, Thermo Scientific, USA), and isolated RNA was left at -80ºC till the manipulation. The reverse transcription step to create complementary DNA (cDNA) was applied by (RevertAid First Strand cDNA Synthesis kits, Thermofisher Scientific, USA). Initially, cDNA synthesis was composed of 1 µl of nuclease-free H_2_O, 1 µl of RT Random Hexamer primer, and 10 µL of extracted RNA that were incubated at 65°c for 5min and cooled on ice then, followed by adding 4µl of 5x RT Buffer, 2 µl of 10Mm dNTP Mix, 1 µl of RevertAid M-MuLV RT Reverse Transcriptase and 1 µl of RiboLock RNase Inhibitor, to reach 20 µl total reaction. The mixture was incubated for 5 min at 25°c operating Applied Biosystems 2720 thermal cycler (Singapore), and cycling settings were boosted for cDNA at 42°c for 60 min, then at 70°c for 5 min. Finally, cDNA count was performed consuming SYBR green-based qRT-PCR by (SensiFAST TM SYBR Lo- ROX Kit, Thermofisher Scientific, Applied Biosystem), using the following designed primers (Thermofisher Scientific, Invitrogen, USA): NEAT1: Forward 5'-TGGCTAGCTCAGGGCTTCAG-3'; Reverse 5'-TCTCCTTGCCAAGCTTCCTTC-3'; TUG1: Forward 5'-TAGCAGTTCCCCAATCCTTG-3'; Reverse 5'-CACAAATTCCCATCATTCCC-3'; IL-6: Forward 5'-CCAGCTATGAACTCCTTCTC-3'; Reverse 5'-GCTTGTTCCTCACATCTCTC-3'; CCL2: Forward 5'-CAGCCAGATGCAATCAATGCC-3'; Reverse 5'-TGGAATCCTGAACCCACTTCT-3'; TNF-α: Forward 5'-CCTCTCTCTAATCAGCCCTCTG-3'; Reverse 5'-GAGGACCTGGGAGTAGATGAG-3'. RNAs and LncRNAs expression were standardized by consuming glyceraldehyde 3-phosphate dehydrogenase (GAPDH) as a housekeeping gene Forward 5'-CTCTGCTCCTCCTGTTCGAC-3'; Reverse 5'-TTAAAAGCAGCCCTGGTGAC-3'. For (qRT-PCR) amplification, we performed a total 20µl reaction (10 µl of Syber green master mix with low ROX dye, 1 µl of forwarding primer and 1 µl of reverse primer of both target and reference gene, 3 µl of cDNA product, 5µl of nuclease-free H_2_O) operating (Applied BioSystems 7500 software version 2.0.1). The cycling program was as follows: initial denaturation for 10 minutes at 95°c, 45 cycles of denaturation for 15 sec at 95°c, and annealing/extension for 1 minute at 60 °c. The comparative ∆∆Ct method was used to achieve relative quantification (RQ) of RNA and lncRNA gene expression **(Supplementary Fig. 1A)**. The focused RNA and lncRNA were standardized to the GAPDH gene and compared to the control. Melting curve analysis was achieved for each gene **(e.g., Supplementary Fig. 1B for NEAT1, Fig 1C for TUG1, Fig 1D for IL-6)** to verify the specificity and lack of primer dimers.

### Statistical methods

Analyzing and tabulating the collected data was done on an IBM personal computer using the Statistical Package of Social Science (SPSS, version 20; SPSS Inc., Chicago, Illinois, USA). The Chi-square test was used to examine the relationship between qualitative variables. The Mann-Whitney test was used for comparing two groups non-normally distributed. For comparing the three groups, we used one-way analysis of variance (ANOVA) and the Kruskal-Wallis test for normally and non-normally distributed quantitative data, respectively. Pearson correlation analysis was used to determine the correlation between the studied biomarkers and the measured laboratory parameters. A receiver operating characteristic (ROC) was applied to evaluate the diagnostic performance of each marker, and a logistic regression analysis was applied to identify the most important independent influencing factors for COVID-19 infection. The significance of the obtained results was decided at the 5% level.

## Results

This study was conducted on 80 COVID-19 patients categorized into two groups: the severe COVID-19 group included 35 patients with severe COVID-19 infection, the moderate COVID-19 group included 45 patients with moderate COVID-19 infection. The control group comprised of 40 participants of matched age and gender who did not have an acute infection and tested negative for COVID-19.

**Table 1** lists their sociodemographic, clinical, and radiological characteristics. Age, sex, and smoking status were not significantly different between the three groups (P>0.05). Comorbid diseases were significantly different between groups (P0.001), with the majority of patients (62.9%) with severe COVID-19 having variable comorbidities such as diabetes (DM), hypertension (HTN), hepatic, renal, and cardiac involvement, compared to patients with moderate COVID-19 and the controls (15.6% and 12.5%, respectively). According to the categorical CT finding, the level of suspicion of infection with COVID-19 ranges from very low or CO-RADS 1 to very high, i.e., CO-RADS 5 and CO-RADS 6 if confirmed with positive RT-PCR. Most patients in the severe and moderate COVID-19 groups had very high suspicion of infection with COVID-19, and ten patients (28.6%) in the severe COVID-19 group and 25 patients (55.6%) in the moderate COVID-19 group had CO-RADS 6. Regarding survival outcomes, all patients in the moderate COVID-19 group (100%) survived for a 12-month follow-up period, whereas 29 patients (82.9%) survived, and six patients (17.1%) died in the severe COVID-19 group (P<0.001).

The laboratory parameters of the studied groups are described in **Table 2**. The severe COVID-19 group had the lowest oxygen concentration, while controls had the highest oxygen concentration. Ferritin levels, D-dimer, and the inflammatory biomarker CRP were significantly higher in the severe and moderate COVID-19 groups compared to controls (P<0.001). In contrast, the total leukocytic count (TLC), hemoglobin (Hb), and absolute lymphocytes were not significantly different between the groups (P>0.05).

**Table 3** shows the measured biomarkers among groups. In COVID-19 patients, we observed predominant upregulation of lncRNAs and cytokine storm. NEAT1 expression was markedly upregulated in the severe COVID-19 group than in controls (P<0.001), with a hundred-fold difference, while no significant difference was observed between the severe and moderate COVID-19 groups (P=0.05). Moreover, NEAT1 expression was higher in the moderate COVID-19 group than in the controls (P<0.001). Regarding TUG1 expression, it was significantly higher in the severe COVID-19 group than in the controls (P<0.001), with a thousand-fold difference, whereas the difference between the severe and moderate COVID-19 groups was not significant (P=0.92). Furthermore, TUG1 expression was remarkably high in the moderate COVID-19 group compared to controls (P<0.001).

In terms of chemokine and cytokine expression in COVID-19 patients, we observed a chemokine and cytokine storm. There was a marked upregulation of CCL2 and IL-6 expression in both severe and moderate COVID-19 groups (P<0.001), with thousands-fold difference compared with controls. The severe COVID-19 group showed significantly higher CCL2 and IL-6 expression levels than the moderate COVID-19 group (P<0.001). Furthermore, TNF-α expression was significantly higher in the severe and moderate COVID-19 groups than in controls (P<0.001), and this increase was less than tenfold higher than in controls. TNF-α expression, on the other hand, differed between the two COVID-19 groups insignificantly (P = 0.13).

The ROC curve was established to distinguish between COVID-19 patients and controls, whereas it detected studied biomarkers' high sensitivity and specificity values. Therefore, they can be considered effective diagnostic biomarkers for the early detection of COVID-19 susceptibility. IL-6 at the cutoff of 0.359 is an efficient diagnostic biomarker with 100% sensitivity, specificity, PPV, NPV, and accuracy, followed by TUG1 at the cutoff of 2.28 with a sensitivity of 98.8%, specificity of 100%, PPV of 100%, NPV of 97.6% and 99.2% accuracy. Moreover, NEAT1 at the cutoff of 90.88 could also be used effectively in COVID-19 susceptibility with 96.3% sensitivity, 100% specificity, 100% PPV, 93% NPV, and 97.5% accuracy. Regarding CCL2, it had 93.8% sensitivity, 100% specificity, 100% PPV, 88.9% NPV, and 95.8% accuracy. Finally, TNF-α had the lowest sensitivity (91.3%), specificity (92.5%), PPV (96.1%), NPV (84.1%), and accuracy (91.7%) **(Table 4) (Fig. 1)**.

Moreover, the roc curve was established to distinguish between the moderate and severe COVID-19, and we found that IL-6 at the cutoff of 356.94 is an efficient determinant of severity with 100% (sensitivity, specificity, PPV, NPV, and accuracy P<0.001), followed by CCL2 at the cutoff of 46654.81 with a sensitivity of 91.1%, specificity of 82.9%, PPV of 87.2%, NPV of 87.9% and 87.5% accuracy P<0.001) and NEAT1 at the cutoff of 1481.008 with (68.9% sensitivity, 68.6% specificity, 73.8% PPV, 63.2% NPV, and 68.8.5% accuracy P=0.05). Nonetheless, TUG1 and TNF-α were not significant biomarkers of severity (P=0.92, P=0.13), respectively **(Table 4)**.

**Figure 2:**
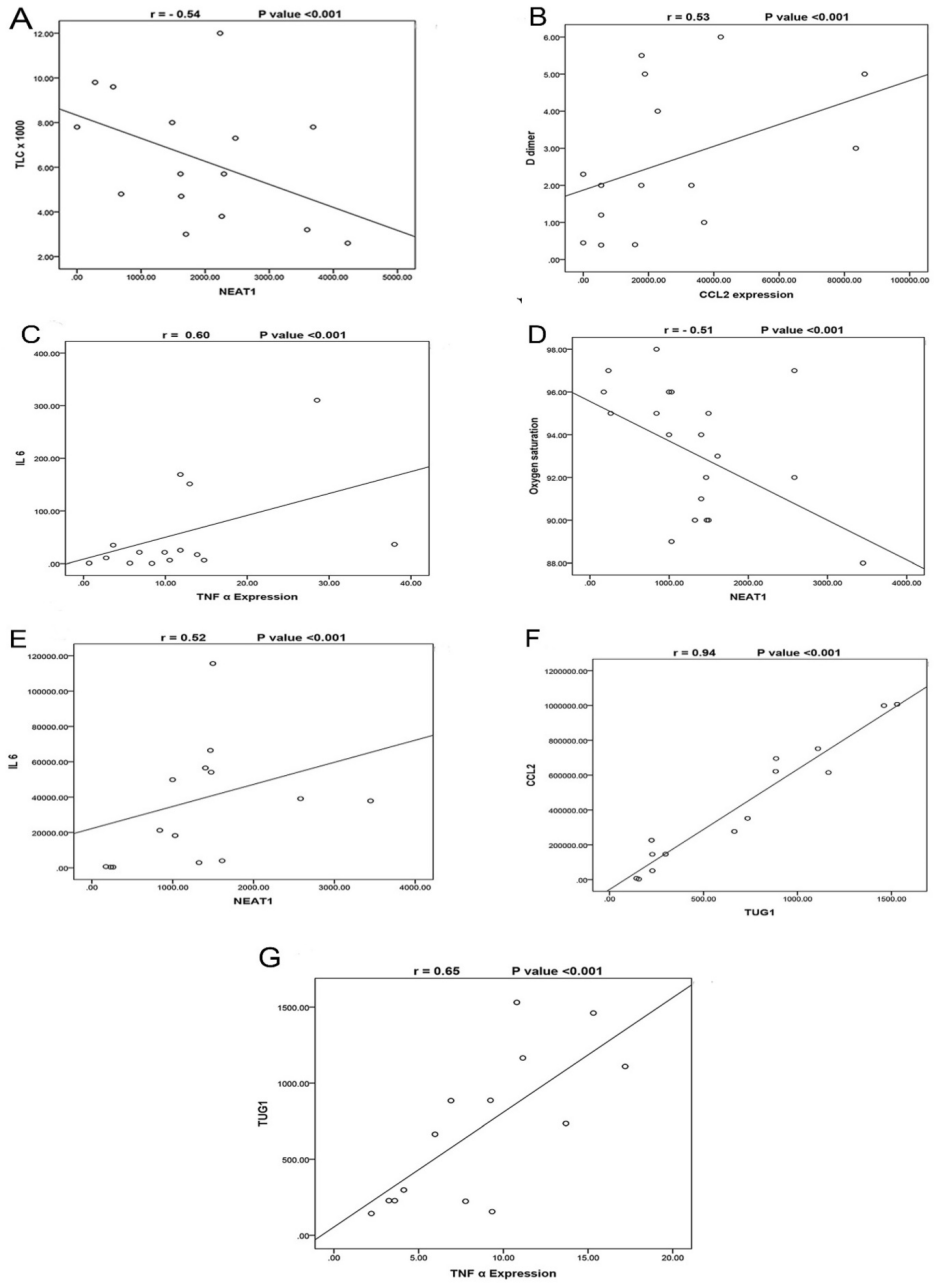
A- Correlation between NEAT1 expression and TLC in the severe COVID-19 group, which shows a negative correlation (N=35) (r= -0.54, p<0.001). B- Correlation between CCL2 expression and D-dimer in the severe COVID-19 group which shows a positive correlation (N=35) (r= 0.53, p<0.001). C- Correlation between TNF-α expression and IL-6 in the severe COVID-19 group, which shows a positive correlation (N=35) (r= 0.60, p<0.001). D- Correlation between NEAT1 expression and Oxygen saturation in the moderate COVID-19 group, which shows a negative correlation (N=45) (r= -0.51, p<0.001). E- Correlation between NEAT1 expression and IL6 expression in the moderate COVID-19 group, which shows a positive correlation (N=45) (r= 0.52, p<0.001). F- Correlation between TUG1 expression and CCL2 expression in the moderate COVID-19 group, which shows a positive correlation (N=45) (r= 0.94, p<0.001). G- Correlation between TUG1 expression and TNF-α expression in the moderate COVID-19 group, which shows a positive correlation (N=45) (r= 0.65, p<0.001).

The correlations between the studied biomarkers and the measured laboratory data are illustrated in **(Table 5)**. In the severe COVID-19 group, we observed that NEAT1 expression was negatively correlated with TLC **(Fig. 2A)** and positively correlated with TUG1 and CCL2 expressions. TUG1 expression was positively correlated with CRP. Furthermore, CCL2 was positively correlated with D-dimer **(Fig. 2B)**, TNF-α, and IL-6 while negatively correlated with TLC and hemoglobin. TNF-α expression was negatively correlated with oxygen and absolute lymphocytes and positively correlated with ferritin, D-dimer, and IL-6 expression **(Fig. 2C)**. In the moderate COVID-19 group, NEAT1 was negatively correlated with oxygen **(Fig. 2D)** but positively with age, TUG1 expression, CCL2, TNF-α, and IL-6 expression **(Fig. 2E)**. TUG1 expression was negatively correlated with oxygen and hemoglobin but positively with CCL2 expression **(Fig. 2F)** and TNF-α **(Fig. 2G)**. CCL2 expression was negatively correlated with oxygen, hemoglobin, and absolute lymphocytes, while it was positively correlated with CRP and TNF-α. A negative correlation was detected between TNF-α and hemoglobin. IL-6 expression was negatively associated with age and oxygen while positively associated with ferritin, CRP, and D-dimer.

**Figure 3:**
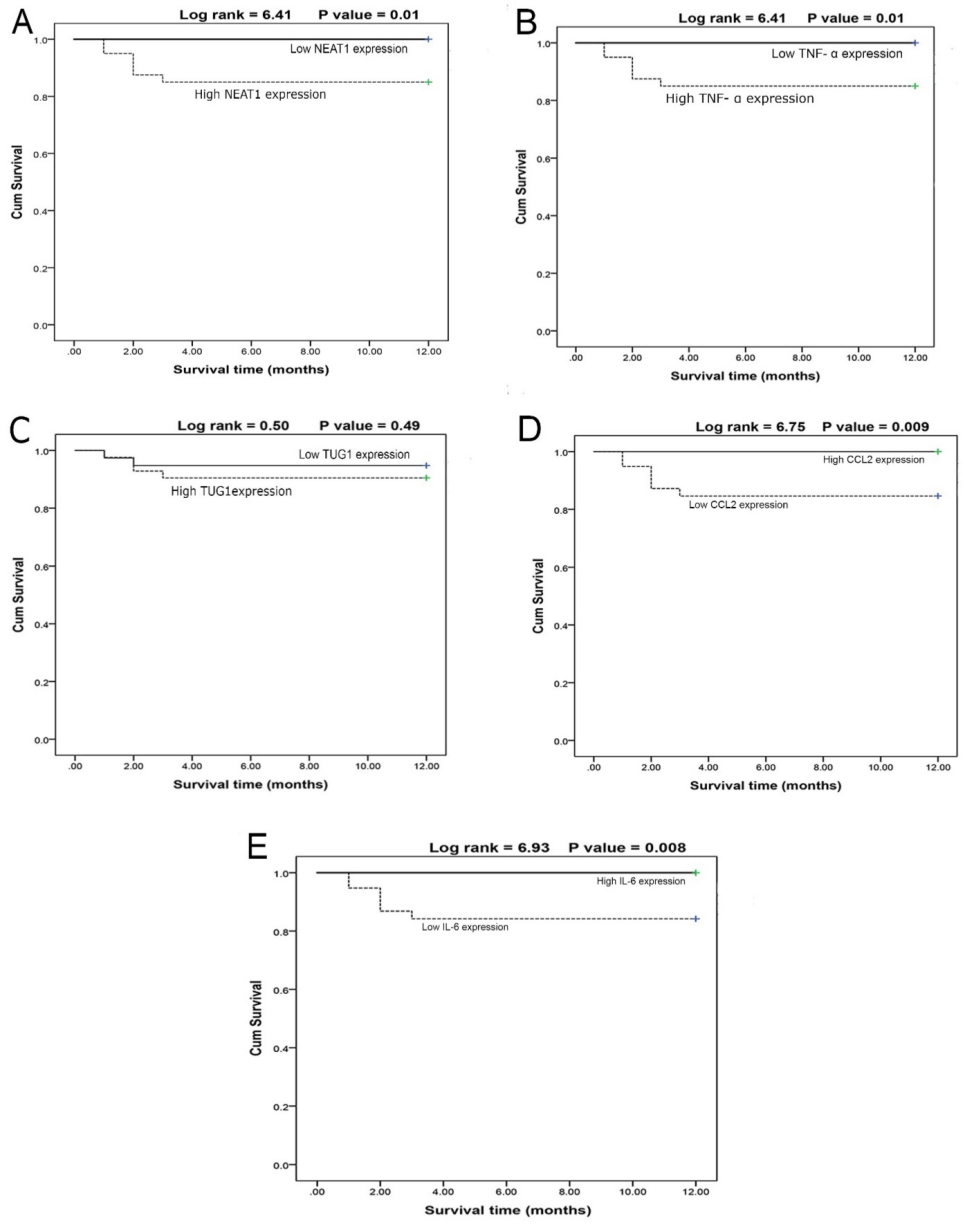
A- Survival curve for cases according to NEAT1 expression (N = 80) (log rank =6.41, p = 0.01). B- Survival curve for cases according to TNF-α expression (N=80) (log rank =6.41, p=0.01). C- Survival curve for cases in relation to TUG1 expression (N = 80) (log rank = 0.50, p = 0.49). D- Survival curve for cases in relation to CCL2 expression (N = 80) (log rank = 6.75, p = 0.009). E- Survival curve for cases in relation to IL-6 value (N = 80) (log rank = 6.93, p = 0.008).

The survival curves of COVID-19 patients concerning the NEAT1 **(Fig. 3A)** and TNF-α **(Fig. 3B)** levels revealed that the high-expression group had lower survival than the low-expression group, whereas TUG1 expression **(Fig. 3C)** had no effect on survival between high and low-expression groups. Moreover, CCL2 **(Fig. 3D)** and IL-6 **(Fig. 3E)** values demonstrated that the high-expression group had higher survival than the low-expression groups. Furthermore, male patients had higher survival rates (P<0.01) than females, and non-smokers had higher survival rates (P<0.001) than smokers.

Multivariate COX regression analysis revealed that NEAT1 is an independent predictor for survival among COVID-19 patients with an odds ratio of 7.48 and 95% CI (1.03 - 36.08) (P=0.02) while (male sex, smokers, CCL2, TNF-α, and IL-6) were cofactors affecting overall survival** (Table 6)**.

## Discussion

Cytokine storm, defined as the hyper-induction of proinflammatory cytokines combined with virus levels, is thought to be a significant feature of severe COVID-19 disease that hastens acute respiratory distress syndrome (ARDS) 29, 30. The severity of COVID-19 disease has been found to be due to the cytokine storm generated by the host immune response, which is an essential aspect of COVID-19 disease immunopathogenesis 31.

Notably, many lncRNAs can control the transcription of cytokines. Recently, 22 lncRNAs were distinguished by targeting ten cytokines upregulated in COVID-19 infection 32. Since lncRNA interferes with inflammatory cytokines, the virus's T cell immune response may be dependent on lncRNA regulation 33. LncRNAs can positively control the differentiation of lymphocytes and T cells closely related to TNF and IL1B 34.

The current study investigated chemokine and cytokine expressions in COVID-19 infection, and we observed chemokine and cytokine storms in COVID-19 patients. We observed CCL2 and IL-6 overexpression in both severe and moderate COVID-19 groups that exceeded thousands of folds compared to controls, and these elevations were significantly higher in the severe COVID-19 group than in the moderate group. Moreover, TNF-α expression was upregulated in both moderate and severe COVID-19 patients compared to controls, and this elevation was less than ten folds.

In addition to exacerbating ARDS in patients with COVID-19, cytokine storms can contribute to tissue damage with subsequent septic shock as well as multi-organ failure 31, 35, 36. Numerous studies have found that cytokines such as IL-2, IL-6, IL-8, IL-10, and TNF-α are overexpressed in COVID-19 infection 37-40. TNF-α and IL-6 levels were higher in patients with moderate COVID-19, while these cytokines were abundant in patients with severe COVID-19 41.

As it induces inflammation, immune responses, and hematopoiesis, IL-6 plays a significant role in the acute host response to infection. A prolonged increase of IL-6 retains chronic inflammation and autoimmunity 42. Clinical trials for the IL-6 antidot tocilizumab drug are currently underway, as IL-6 has recently been investigated as a potential drug target 38, 43-45.

TNF-α is the main trigger for cytokine storms, provoking the changeover from inflammation to fibrosis in the lungs. The proinflammatory cytokine TNF-α is implicated in lung and vascular tissue damage as well as ARDS and blood clotting disorders 46, 47. CCL2, also termed monocyte chemoattractant protein 1 (MCP-1), is a chief promoter of fibrosis and is believed to be an anti-fibrotic drug target 48. Prior research showed that CCL2 facilitates the differentiation and recruitment of fibroblasts to the alveolar space, aggravating much of the collagen deposition. CCL2 endorsed fibroblast survival and provoked IL-6 synthesis 49. Notably, CCL2, IL-1, IL-6, and TNFα also regulate fibrosis 48, and their down-regulation might serve as a prospective anti-fibrotic effect.

**Sierra et al**. illustrated that CCL2 expression was upregulated in patients with an unfavorable outcome and was related to severe asthenia and dyspnea 50.

In terms of lncRNA expression, we found that NEAT1 was significantly overexpressed in both severe and moderate COVID-19 patients compared to controls, and this difference exceeded hundreds of folds. With respect to the TUG1 expression, it was substantially elevated in both severe and moderate COVID-19 patients, more than a thousand-fold higher than the controls. This striking overexpression of NEAT1 and TUG1 in severe and moderate COVID-19 patients may explain the prominent role of lncRNAs in COVID-19 and may augment their use as targets for control and downregulation in the management of COVID-19 infection.

NcRNAs represent the transcribed but untranslated part of the genome. Ongoing studies have demonstrated their association with the immune response to SARS-CoV-2 and the development of COVID-19, controlling the cytokine storm, hemostatic variation, immune cell mobilization, and vascular dysregulation. The lncRNAs are prospective tools for stratifying disease, directing SARS-CoV-2-originating RNA into changing the behavior of the host's RNA, proteins, and signaling pathways, thereby altering the outcome of infection 51.

Based on lncRNA transcriptome data collected from SARS-CoV-2 infected bronchial epithelial cells, Vishnubalaji et al. discovered upregulation of NEAT1 and the metastasis-associated lung adenocarcinoma transcript 1 (MALAT1) 52. Furthermore, overexpression of NEAT1 and MALAT1 in COVID-19 patients was also isolated from bronchoalveolar lavage fluid 10. Additionally, elevated NEAT1 expression in peripheral blood mononuclear cells was detected in patients with severe COVID-19 rather than in those with moderate disease and healthy subjects 5. Interestingly, a single-cell transcriptomic study in the respiratory tract and the peripheral blood of patients with mild and severe COVID-19 has revealed that NEAT1 and MALAT1 are differentially expressed 5.

In SARS-CoV-2 positive individuals, Rodrigues et al. 53 discovered overexpression of NEAT1 and MALAT1 in saliva and nasopharyngeal samples, which may be linked to the early response to COVID-19 infection, viral dosage, and clinical manifestations. Furthermore, they added that NEAT1 and MALAT1 are vital antiviral immune response components.

TUG1 expression has been linked to a variety of diseases in previous research. Previous research, in particular, revealed a direct relationship between TUG1 knockdown and a decline in the inflammatory response in atherosclerosis 54. Qiu et al. 55 showed that TUG1 repressed apoptosis and production of TNF-α, IL-1β, and IL-6 in lipopolysaccharide-treated pulmonary microvascular endothelial cells that were reestablished using miR-34b-5p mimics, in acute lung injury. TUG1 also contributes to cerebral ischemia and reperfusion injury by sponging miR-493-3p or miR-410-3p and activating the JNK and p38 MAPK pathways 56.

A previous study showed that TUG1 was downregulated in non-small cell lung cancer 15. TUG1 siRNA promotes the proliferation of human lung epithelial cells (BEAS-2B) and fibroblasts (HFL1), and TUG1 deletion reduces the production of alpha-smooth muscle actin (α-SMA) and fibronectin proteins which are mesenchymal markers in BEAS-2B and HFL1 cells 57.

The most outstanding findings in this study are the significant correlations between NEAT1 and TUG1 and measured cytokines with other laboratory data, including oxygen concentration, D-dimer, ferritin, total leukocytic count, hemoglobin, and absolute lymphocytes. These findings might partially explain the prominent role of lncRNAs in moderate and severe COVID-19 infections as they can regulate the secretion of many cytokines that can cause severe inflammation and precipitate ARDS in COVID-19.

Furthermore, we identified significant positive correlations between NEAT1 and CCL2, and IL-6, which may explain its pathological role in COVID-19 by targeting CCL2 and IL-6 and increasing their production, specifically in the severe form of the disease. Furthermore, TUG1 had a positive correlation with CCL2 and TNF-α, implying that TUG1 is involved in CCL2 and TNF-α production, which explains its role in COVID-19. In addition, we detected positive correlations between NEAT1 and TUG1, which supports the interaction of lncRNAs that can trigger a cascade of cytokines that further increase the severity of the disease.

Morenikeji et al. pinpointed that TUG1 focuses on two of the key cytokines, IL-7 and CCL2, during COVID-19 infection 32. NEAT1 has been involved in inflammatory cytokines regulation, including IL-6, IL-1β, and TNF-α, and ultimately neuropathic pain in the chronic constriction injury (CCI) rats' model. Inhibition of NEAT1 can induce miR-381 expression and consequently down-regulation of inflammatory cytokines such as IL-6, IL-1β, and TNF-α, which suggests the crucial role of NEAT1 in the regulation of IL-6 expression 58. NEAT1 is a crucial activator of the NLRP3 inflammasome and the NLRC4 and AIM2 inflammasomes that consequently amplify the inflammatory response 59.

Because lncRNAs are transcriptionally and epigenetically regulated, they can target critical cytokine nucleotide sequences, regulating cytokine expression and altering the immune response to COVID-19 infection, thus promoting future use of antisense oligonucleotide knockdown, RNAi knockdown, and viral gene therapy 60-62.

Limitations of the study include small sample size, single-center study, variable clinical presentation, and not all strains of COVID virus were included in the study. We suggest a further multicenter study with larger sample size and with the use of RNA interference therapy to validate our results.

## Conclusion

In this study, marked distinguishable overexpression of lncRNAs NEAT1 and TUG1**,** and a remarkable cytokine storm in both moderate and severe forms of COVID-19 infection are observed. Furthermore, significant correlations were detected between lncRNAs and IL-6, CCL2, TNF-α, and other diagnostic biomarkers that might explain the pathogenic role of NEAT1 and TUG1 in disease through targeting the production of these cytokines, drawing attention to the use of RNAi knockdown for lncRNAs under expression that may partially put off the cytokine storm in COVID-19 infection and preclude ARDS.

## Supplementary Material

Supplementary figure.

## Figures and Tables

**Table 1:** Socio-demographic, clinical, and radiological characteristics of the studied groups

	Severe COVID-19 Group N = 35	Moderate COVID-19 Group N = 45	Control Group N = 40	Test	P-value
**Age**				F 2.13	0.12
Mean ±SD	51.37±10.24	45.84±11.54	48.05±13.56		
Median (Range)	53 (37 - 70)	44 (27 - 69)	47 (25 - 72)		
**Sex**				X^2^ 0.61	0.74
Male	20 (57.1)	22 (48.9)	22 (55.0)		
Female	15 (42.9)	23 (51.1)	18 (45.0)		
**Smoking**				X^2^ 1.38	0.50
Yes	12 (34.3)	11 (24.4)	14 (35.0)		
No	23 (65.7)	34 (75.6)	26 (65.0)		
**Comorbidity**				X^2^ 29.10	<0.001
Yes	22 (62.9)	7 (15.6)	5 (12.5)		
No	13 (37.1)	38 (84.4)	35 (87.5)		
**Comorbidity**				X^2^ 34.20	<0.001
No	13 (37.1)	38 (84.4)	35 (87.5)		
DM	7 (20.0)	4 (8.9)	3 (7.5)		
HTN	9 (25.7)	3 (6.7)	2 (5.0)		
Hepatic	2 (5.7)	0 (0.0)	0 (0.0)		
Renal	2 (5.7)	0 (0.0)	0 (0.0)		
Cardiac	2 (5.7)	0 (0.0)	0 (0.0)		
Fever	35 (100)	35 (77.8)	0 (0.0)	88.0	<0.001
Dyspnea	33 (94.3)	21 (46.7)	0 (0.0)	67.12	<0.001
Cough	32 (91.4)	32 (71.1)	0 (0.0)	71.84	<0.001
Tachycardia	35 (100)	0 (0.0)	0 (0.0)	120	<0.001
Wheezy	13 (37.1)	6 (13.3)	0 (0.0)	19.66	<0.001
Crepitation	18 (51.4)	6 (13.3)	0 (0.0)	32.86	<0.001
**CT.CO-RADS**				X^2^ 5.901	0.052
1	0 (0.0)	0 (0.0)			
2	0 (0.0)	0 (0.0)			
3	0 (0.0)	0 (0.0)			
4	9 (25.7)	8 (17.8)			
5	16 (45.7)	12 (26.7)			
6	10 (28.6)	25 (55.6)			
**Outcome**				X^2^ 8.34	<0.001
Survived	29 (82.9)	45 (100)			
Died	6 (17.1)	0 (0.0)			

F = F test of ANOVA, X^2^ = Chi-square test, SD = Standard deviation, DM; diabetes mellitus, HTN; hypertension, CO-RADS; COVID-19 Reporting and Data System

**Table 2:** Laboratory values of the studied groups.

	Severe COVID-19 Group N = 35	Moderate COVID-19 Group N = 45	Control Group N = 40	Test	P-value
**Oxygen concentration %**				F 372.5	<0.001
Mean ±SD	80.83±3.68	85.0±3.03	98.93±0.64		
Median (Range)	80 (75 - 86)	85 (80- 90)	98.0 (97 - 99)		
**Oxygen concentration value**				X^2^ 76.75	<0.001
Low	35 (100%)	27 (60%)	0 (0.0%)		
Normal	0 (0.0%)	18 (40.0%)	40 (100%)		
**Ferritin (ng/ ml)**				K 5.14	<0.001
Mean ±SD	552.66±483.67	113.13±54.72	82.5±33.38		
Median (Range)	500 (71 - 2000)	99 (60 - 300)	76.5 (40 - 200)		
**Ferritin value**				X^2^ 68.08	<0.001
Normal	10 (28.6%)	43 (95.6%)	40 (100%)		
High	25 (71.4%)	2 (4.4%)	0 (0.0%)		
**CRP (mg/L)**				K 5.87	<0.001
Mean ±SD	56.26±23.89	23.27±9.11	3.18±1.08		
Median (Range)	57 (20 - 90)	22 (6 - 40)	3 (1 - 5)		
**D -dimer (µg/ml)**				K 6.40	<0.001
Mean ±SD	2.71±1.83	0.46±0.25	0.37±0.06		
Median (Range)	2 (0.39 - 6)	0.40 (0.22 - 1.2)	0.37 (0.28 - 0.48)		
**TLC (x10^3^)**				K 0.19	0.85
Mean ±SD	6.49±2.73	6.43±2.01	8.95±1.70		
Median (Range)	5.7 (2.6 - 12.0)	6 (2.6 - 9.6)	8.7 (6.3 - 11.4)		
**Hb (gm/dl)**				F 0.68	0.50
Mean ±SD	11.84±1.61	12.06±1.31	12.15±1.22		
Median (Range)	11.9 (9.1 - 15.9)	12.3 (9 - 14.4)	12.05 (8.5 - 14)		
**Absolute lymphocytes**				K 1.65	0.10
Mean ±SD	1.38±0.92	1.71±0.85	2.16±0.54		
Median (Range)	1.6 (0.19 - 2.9)	1.8 (0.32 - 3.0)	2.2 (1.1 - 3)		

F = F test of ANOVA K = Kruskal Wallis test CRP; C-reactive protein, TLC; total leukocytic count, Hb; hemoglobin High: elevated above the normal reference value

**Table 3:** Measured biomarkers of the studied groups.

	Severe COVID-19 Group N = 35	Moderate COVID-19 Group N = 45	Control Group N = 40	U	P-value
**NEAT1 Expression**				1.95	0.05^1^
Median	1626.59	1404.87	2.20	6.26	<0.001^2^
(Range)	(0.55 - 4225.06)	174.79 - 3448.12.	0.44 - 6.97	7.93	<0.001^3^
**TUG1 Expression**				0.10	0.92^1^
Median	623.11	664.15	0.56	7.44	<0.001^2^
(Range)	3.0 - 1554.18	143.03 - 1530.52	0.12 - 1.57	7.93	<0.001^3^
**CCL2 Expression**				6.44	<0.001^1^
Median	276678.7	17876.56	2.36	7.93	<0.001^2^
(Range)	2951.78 - 1007200	0.45 - 86210.41	5.01 - 7.84	5.32	<0.001^3^
**TNF-α Expression**				1.52	0.13^1^
Median	11.83	7.77	1.35	6.48	<0.001^2^
(Range)	0.68 - 38	2.21 - 17.19	0 - 3.73	7.52	<0.001^3^
**IL-6 Expression**				7.65	<0.001^1^
Median	37877.43	21.33	0.02	7.93	<0.001^2^
(Range)	403.66 - 115591.9	0.47 - 310.22	0- 0.25	7.44	<0.001^3^

U = Mann-Whitney U test 1 = comparing severe and moderate COVID-19 groups 2 = comparing severe COVID-19 group and controls 3 = comparing moderate COVID-19 group and controls

**Table 4:** ROC curve analysis of the studied markers to differentiate between COVID-19 patients and control and for discrimination of severity.

	**Differentiation between total patients and control**				
	**NEAT1 Expression**	**TUG1 Expression**	**CCL2 Expression**	**TNF-α Expression**	**IL-6 Expression**
**AUC**	0.965	0.999	0.938	0.957	1.0
**P value**	<0.001	<0.001	<0.001	<0.001	<0.001
**95% CI**	0.93 - 1.0	0.997 - 1.0	0.88 - 0.99	0.93 - 99	1.0 - 1.0
**Cutoff point**	90.88	2.28	0.839	3.245	0.359
**Sensitivity**	96.3%	98.8%	93.8%	91.3%	100%
**Specificity**	100%	100%	100%	92.5%	100%
**PPV**	100%	100%	100%	96.1%	100%
**NPV**	93.0%	97.6%	88.9%	84.1%	100%
**Accuracy**	97.5%	99.2%	95.8%	91.7%	100%
	**Differentiation between moderate and severe COVID-19 patients**				
	**NEAT1 Expression**	**TUG1 Expression**	**CCL2 Expression**	**TNF-α Expression**	**IL-6 Expression**
**AUC**	0.63	0.51	0.92	0.60	1.0
**P value**	0.05	0.92	<0.001	0.13	<0.001
**95% CI**	0.49 - 0.76	0.38 - 0.64	0.86 - 0.99	0.47 - 0.73	1.0 - 1.0
**Cutoff point**	1481.008	643.63	46654.81	9.62	356.94
**Sensitivity**	68.9%	60.0%	91.1%	64.4%	100%
**Specificity**	68.6%	57.1%	82.9%	62.9%	100%
**PPV**	73.8%	64.3%	87.2%	69.0%	100%
**NPV**	63.2%	52.6%	87.9%	57.9%	100%
**Accuracy**	68.8%	58.8%	87.5%	51.3%	100%

AUC; area under the curve, PPV; positive predictive value, NPV; negative predictive value.

**Table 5:** Correlation between the studied biomarkers and the other measured laboratory parameters in COVID-19 patients' groups.

	**Severe COVID-19 Group**									
	**NEAT1 Expression**		**TUG1 Expression**		**CCL2 Expression**		**TNF -α Expression**		**IL-6 Expression**	
	r	P-value	r	P-value	r	P-value	r	P-value	r	P-value
**Age**	0.25	0.15	0.01	0.96	0.20	0.24	-0.23	0.18	-0.29	0.09
**Oxygen**	-0.11	0.53	0.17	0.35	-0.21	0.22	-0.35	0.04	-0.16	0.37
**Ferritin**	0.18	0.30	0.33	0.05	0.03	0.87	0.47	0.005	0.17	0.32
**CRP**	0.02	0.92	0.37	0.03	0.31	0.07	0.19	0.27	0.08	0.32
**D -dimer**	0.24	0.17	0.28	0.10	0.53	0.001	0.35	0.04	0.32	0.06
**TLC**	-0.54	0.001	-0.21	0.22	-0.35	0.04	-0.24	0.16	0.06	0.75
**Hb**	0.09	0.63	-0.08	0.65	-0.63	<0.001	-0.09	0.61	0.02	0.90
**Absolute lymphocytes**	-0.15	0.39	0.002	0.99	-0.14	0.41	-0.42	0.01	0.12	0.48
**NEAT1 Expression**	---	-----	0.60	<0.001	0.39	0.02	0.13	0.46	0.06	0.73
**TUG1 Expression**	0.60	<0.001	----	-----	0.28	0.11	0.30	0.09	0.30	0.09
**CCL2 Expression**	0.39	<0.02	0.28	0.11	----	-----	0.41	0.01	0.53	0.001
**TNF-α Expression**	0.13	0.46	0.30	0.09	0.41	0.01	----	-----	0.60	<0.001
**IL-6 Expression**	0.06	0.73	0.30	0.08	0.53	0.001	0.60	<0.001	----	-----
	**Moderate COVID-19 Group**									
	**NEAT1 Expression**		**TUG1 Expression**		**CCL2 Expression**		**TNF- α Expression**		**IL-6 Expression**	
	r	P-value	r	P-value	R	P-value	r	P-value	r	P-value
**Age**	0.32	0.03	0.17	0.28	0.08	0.59	0.09	0.58	-0.22	0.045
**Oxygen**	-0.51	<0.001	-0.30	0.04	-0.36	0.02	-0.23	0.12	-0.69	<0.001
**Ferritin**	0.10	0.52	0.12	0.45	0.21	0.17	0.03	0.84	0.44	<0.001
**CRP**	0.04	0.80	0.28	0.06	0.40	0.006	0.24	0.12	0.60	<0.001
**D -dimer**	0.08	0.62	0.057	0.71	0.06	0.68	0.11	0.46	0.61	<0.001
**TLC**	0.16	0.29	0.19	0.20	0.15	0.31	0.02	0.92	0.06	0.60
**Hb**	0.10	0.51	-0.47	0.001	-0.47	0.001	-0.37	0.01	0.11	0.32
**Absolute lymphocytes**	-0.27	0.07	-0.27	0.07	-0.32	0.03	-0.13	0.38	0.16	0.17
**NEAT1 Expression**	----	---	0.57	<0.001	0.63	<0.001	0.38	0.01	0.52	<0.001
**TUG1 Expression**	0.57	<0.001	----	---	0.94	<0.001	0.65	<0.001	-0.05	0.76
**CCL2 Expression**	0.63	<0.001	0.94	<0.001	----	---	0.72	<0.001	0.06	0.72
**TNF-α Expression**	0.38	0.01	0.65	<0.001	0.72	<0.001	----	---	-0.15	0.34
**IL-6 Expression**	0.52	<0.001	0.05	0.76	0.06	0.72	0.15	0.34	----	---

**Table 6:** Cox regression for independent factors affecting overall survival among the studied patients.

	SE	Wald X^2^	P-value	Odds ratio	95.0% CI	
					Lower	Upper
**Sex (Males)**	2.01	1.33	0.19	0.91	0.46	3.55
**Smoking**	1.88	0.99	0.36	1.05	0.31	4.58
**NEAT1**	1.01	5.25	0.02	7.48	1.03	36.08
**CCL2**	1.09	0.20	0.65	1.05	0.33	22.07
**TNF-α**	2.05	0.93	0.33	1.15	0.46	19.03
**IL.6**	1.03	0.41	0.52	0.97	0.41	6.92

SE; standard error, 95%CI; 95% confidence interval.
