# Supplementary material for: Interplay of LncRNAs NEAT1 and TUG1 in Incidence of Cytokine Storm in Appraisal of COVID-19 Infection
Source: Int J Biol Sci. 2022 Jul 18;18(13):4901–13. doi: 10.7150/ijbs.72318 (PMC9379411; doi:10.7150/ijbs.72318)
Supplement: Supplementary file 1 — Supplementary figure. [file ijbsv18p4901s1.pdf]

Supplementary data:

Supplementary figure 1: A- Amplification plot of target genes where RQ of gene expression of RNA and lncRNA, was accomplished by the comparative ΔΔCt method. B- Melting curve analysis of NEAT1 to verify the specificity and lack of primer dimers C-Melting curve analysis of TUG1. D-melting curve analysis of IL-6.

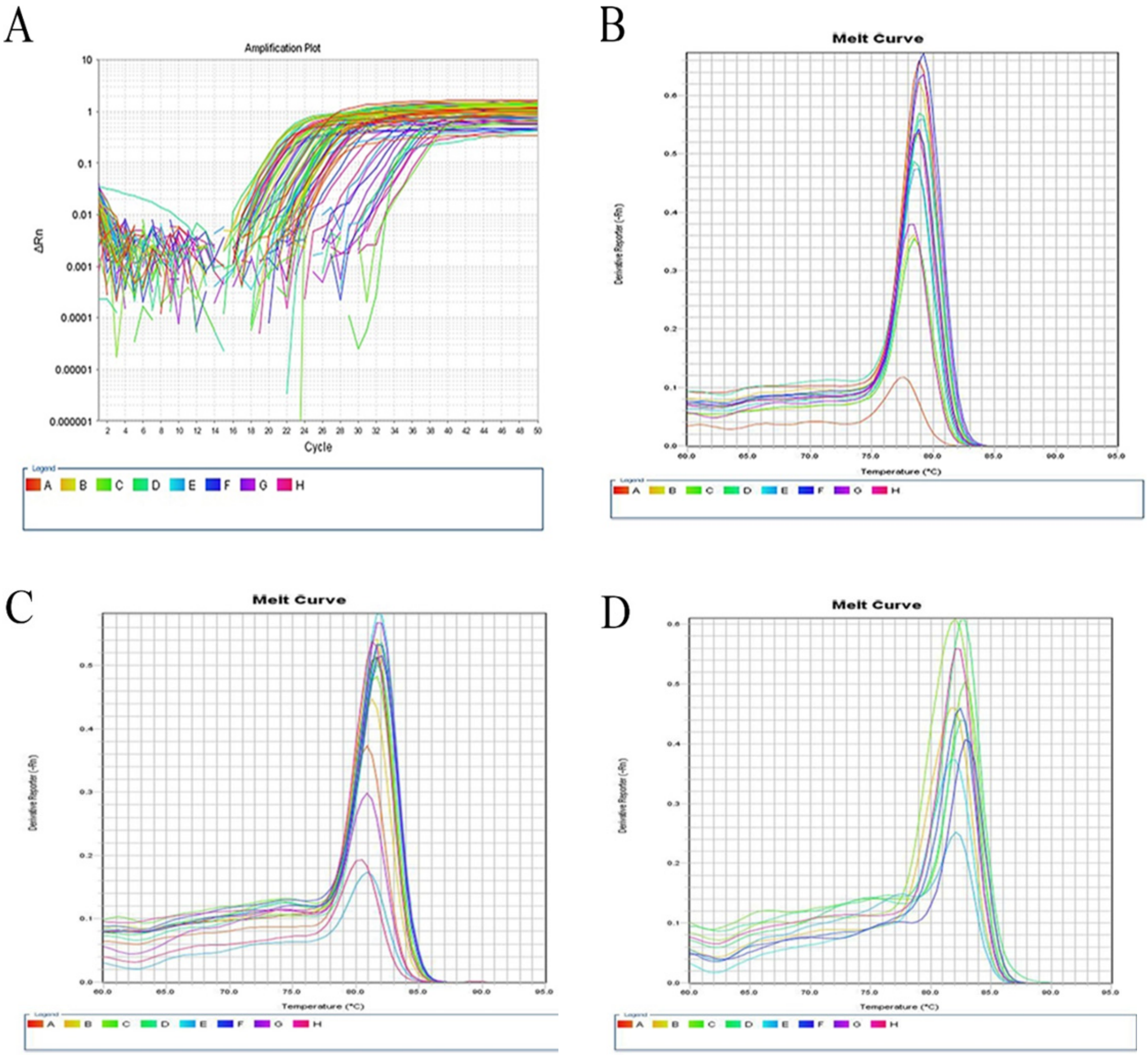

Supplementary figure 1
